# Retinal vascular metrics difference by comparison of two image acquisition modes using a novel OCT angiography prototype

**DOI:** 10.1371/journal.pone.0243074

**Published:** 2020-12-01

**Authors:** Luca Di Antonio, Pasquale Viggiano, Giada Ferro, Lisa Toto, Rossella D’Aloisio, Annamaria Porreca, Marta Di Nicola, Rodolfo Mastropasqua

**Affiliations:** 1 UOC Ophthalmology ASL-1 Avezzano-Sulmona-L’Aquila, L’Aquila, Italy; 2 Department of Medicine and Science of Ageing, Ophthalmology Clinic, University G. d’Annunzio Chieti-Pescara, Chieti, Italy; 3 Department of Medical, Oral and Biotechnological Sciences, Laboratory of Biostatistics, University “G. d’Annunzio” Chieti-Pescara, Chieti, Italy; 4 Department of Ophthalmology, University of Modena and Reggio Emilia, Modena, Italy; University of California Los Angeles, UNITED STATES

## Abstract

**Purpose:**

To assess the different impact of two enface OCTA image simultaneously acquired by means of a new prototype of Spectral-Domain Optical Coherence Tomography Angiography (SD-OCTA) on quantitative retinal vascular metrics.

**Methods:**

In this prospective observational cross-sectional study 28 healthy subjects were enrolled. Macular (3x3 mm) OCTA images were acquired for all participants using Solix Fullrange OCT (Optovue Inc, Freemont CA, USA). The main outcome measurements were: Perfusion density (PD), vessel length density (VLD), and vessel diameter index (VDI) of both superficial capillary plexus (SCP) and deep capillary plexus (DCP), and choriocapillaris (CC) total flow-deficits area. Quantitative retinal vascular metrics were measured on binarized and skeletonized OCTA images by comparing not averaged and fast automated multiple averaged en face OCTA images.

**Results:**

In both SCP and DCP, PD significantly increased (p = 0,005 and p = 0,030, respectively), and VLD significantly decreased (p<0,001 and p = 0,004, respectively), and VDI increased (p<0,001 and p = 0,068, respectively), and total CC flow deficits area significantly decreased (p<0,001) by averaging multiple OCTA images.

**Conclusions:**

In this study, we found a significant difference of quantitative retinal metrics by comparing two different image acquisition modes using a novel and fully automated averaging OCTA system in young healthy subjects.

## Introduction

Optical coherence tomography angiography (OCTA) is a new, fast, safe, and dyeless method, widely spread into clinical practice. It can easily reveal the features of retinal vascular layers that usually are not visualized with other imaging techniques [1]. OCTA is mainly used to image both retinal superficial capillary plexus (SCP) and deep capillary plexus (DCP), and the choriocapillaris (CC) which is the tiny innermost part of the choroid. Moreover OCTA, unlike to the standard Fluorescein Angiography (FA), is able to quantify new functional metrics such as perfusion density (PD), and vessel length density (VLD), and vessel diameter index (VDI) useful for evaluating vascular changes in different retinal diseases and for monitoring their treatment response [2, 3]. Although OCTA is considered to be a new paradigm shift for the retinal assessment, it has some limitations of which image noise and blur that may lead to both erroneous detections of a vessel structure and estimation of quantitative OCTA metrics [4]. Recent studies have shown that averaging multiple en face OCTA images improves both image quality and quantitative metrics, because of it increase the signal-to-noise ratio [5]. This study aimed to evaluate the difference of retinal vascular metrics by comparison of two image acquisition mode using a novel SD-OCTA prototype instrument.

## Methods

In this prospective observational cross-sectional study, healthy volunteers between 21 and 33 years of age were enrolled. The study was performed at the Ophthalmology Clinic of the University G. d’Annunzio, Chieti-Pescara, Italy between December 2019 and February 2020. The study adhered to the tenets of the Declaration of Helsinki and was approved by the Institutional Review Board (IRB) (Department of Medicine and Science of Ageing, University G. d’Annunzio Chieti-Pescara). Informed consent was obtained before the scanning session. All subjects received a comprehensive ophthalmic examination, which included the measurement of best-corrected visual acuity (BCVA) using Early Treatment Diabetic Retinopathy Study chart, slit-lamp biomicroscopy, intraocular pressure (IOP) with Goldmann applanation tonometry, and dilated funduscopic examination using a 78 D (diopters) lens. Inclusion criteria were BCVA of 20/25 or better, spherical refraction within ±3.0 D, and cylinder correction within ±2.0 D. Exclusion criteria ware evidence or history of previous ocular disease, presence of lens opacities, previous surgery, laser or medical treatments, evidence or history of systemic disease with ocular involvement.

### Imaging protocol

**Fig 1:**
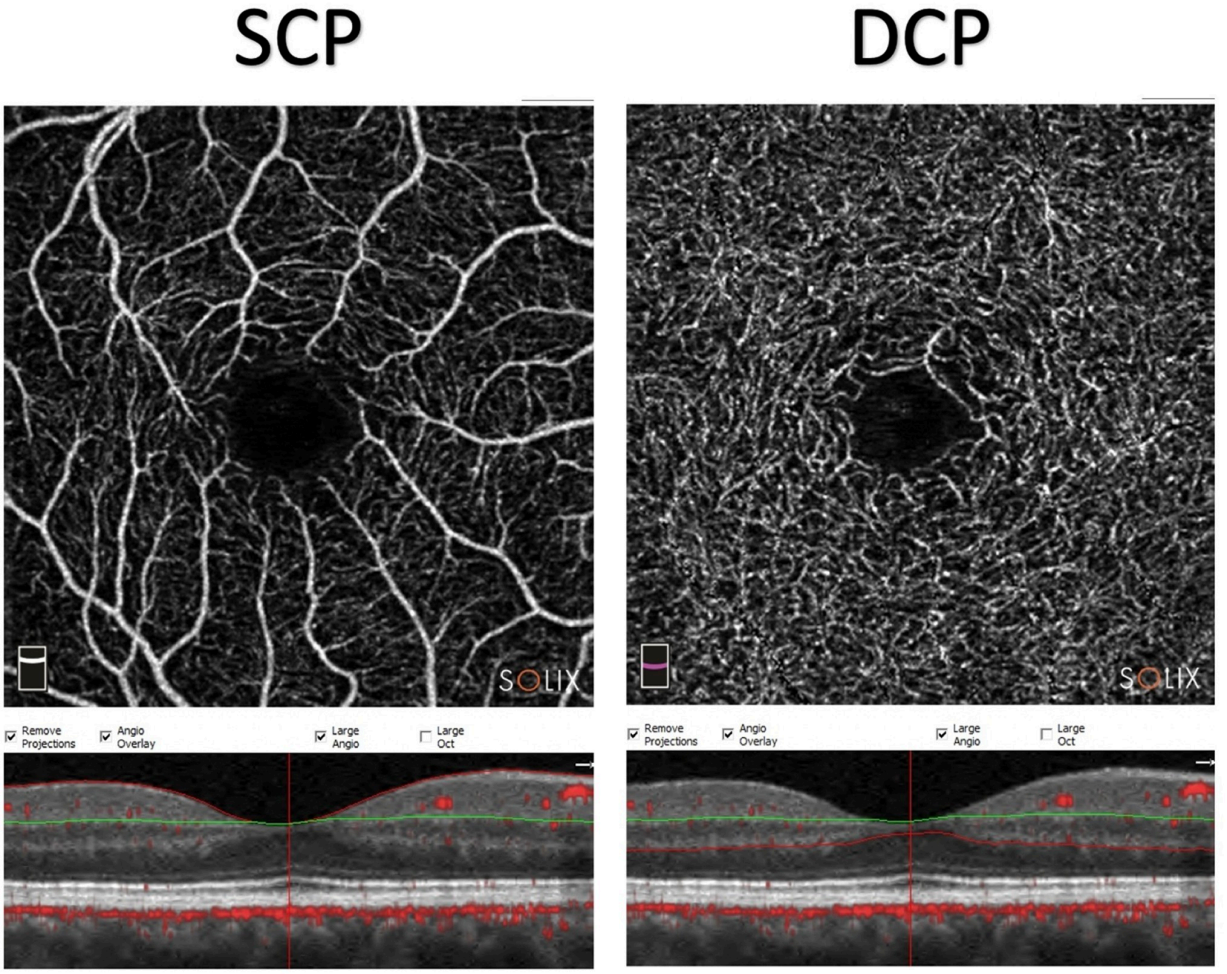
Representation of the structural SD-OCT layers segmentation for superficial capillary plexus (SCP) and deep capillary plexus (DCP).

All subjects were imaged with Solix Fullrange OCT (Optovue Inc, Freemont CA, USA), a new ultra-high-speed SD-OCTA device (version 2019 V1.0.0.305) which operates at 120,000 A-scans per second with the split spectrum amplitude-decorrelation angiography (SSADA) algorithm. This latter, as previously and widely reported, creates a contrast between static and non-static tissue that allows the visualization of the blood flow in the capillary bed by calculating the decorrelation signal amplitude from consecutive B-scans at the same retinal location [6]. The Solix device is able to perform two different OCTA protocol scans: a standard single not-averaged scan volume, and a multi-volume merge average four scan volumes to deliver high-density images with pristine clarity. Before imaging, each subject’s pupils were dilated with a combination of 0.5% tropicamide and 10% phenylephrine. Study participants underwent both scanning protocols, consisting of 3x3 mm (304x304 pixels in the transverse dimension) field of view centered on the fovea. An internal fixation light was used to center the scanning area. The imaging protocol was acquired by a single trained operator by capturing, in one randomly selected eye of each patient, a couple of 3x3 mm en face OCTA angiogram, consecutively: one for single and other for multi-volume scans, respectively. Randomization was achieved using the random number generator Pro 2.17 (free software that is available on https://random-number-generator-pro.soft112.com/). These scans included two volumetric and orthogonal OCT data set for the not averaged en face OCTA images and eight volumetric and orthogonal OCT data set for the averaged en face OCTA images, each one captured in about 2,5 seconds, respectively. After completion of the volumetric OCT data sets, the software applied Motion Correction Technology (MCT), a patented post-processing tool that enables true three-dimensional (3D) correction of distortion in all directions for ultra-precise motion correction. Low-quality scans (i.e., if the subject blinked or the scan had significant motion artifacts) were excluded and repeated until good-quality scans were achieved with a signal strength was ≥8. New segmentation algorithms embedded in the device were used to rightly assess SCP, DCP (Fig 1), and CC layers, as previously reported [7]. The 3D projection artifact removal (PAR) 2.0 was applied to rapidly remove the projection artifact from the DCP and CC to simplify image interpretation ad produce more reliable quantification. Before image processing, two retinal specialists independently (LDA and PV) carefully visualized all selected images to ascertain the correctness of the position of the upper and lower boundaries of segmentation such as the inner limiting membrane (ILM) and retinal pigment epithelium (RPE), respectively. If segmentation errors were present, the user could manually correct few or all affected B-scans and then propagate the correction through a user-selected region or throughout the entire scan volume to enhance the definition of en face OCTA slab for both qualitative and quantitative analysis.

### Image processing

The main outcome measures were: (i) SCP perfusion and vessel length densities; (ii) DCP perfusion and vessel length densities; (iii) SCP and DCP vessel diameter index; (iiii) the total signal void area, which represents a measure of the total area of CC vascular dropout (absence of flow or flow below the slowest detectable threshold) as a percentage of each analyzed area [Flow Deficits(FD)].

**Fig 2:**
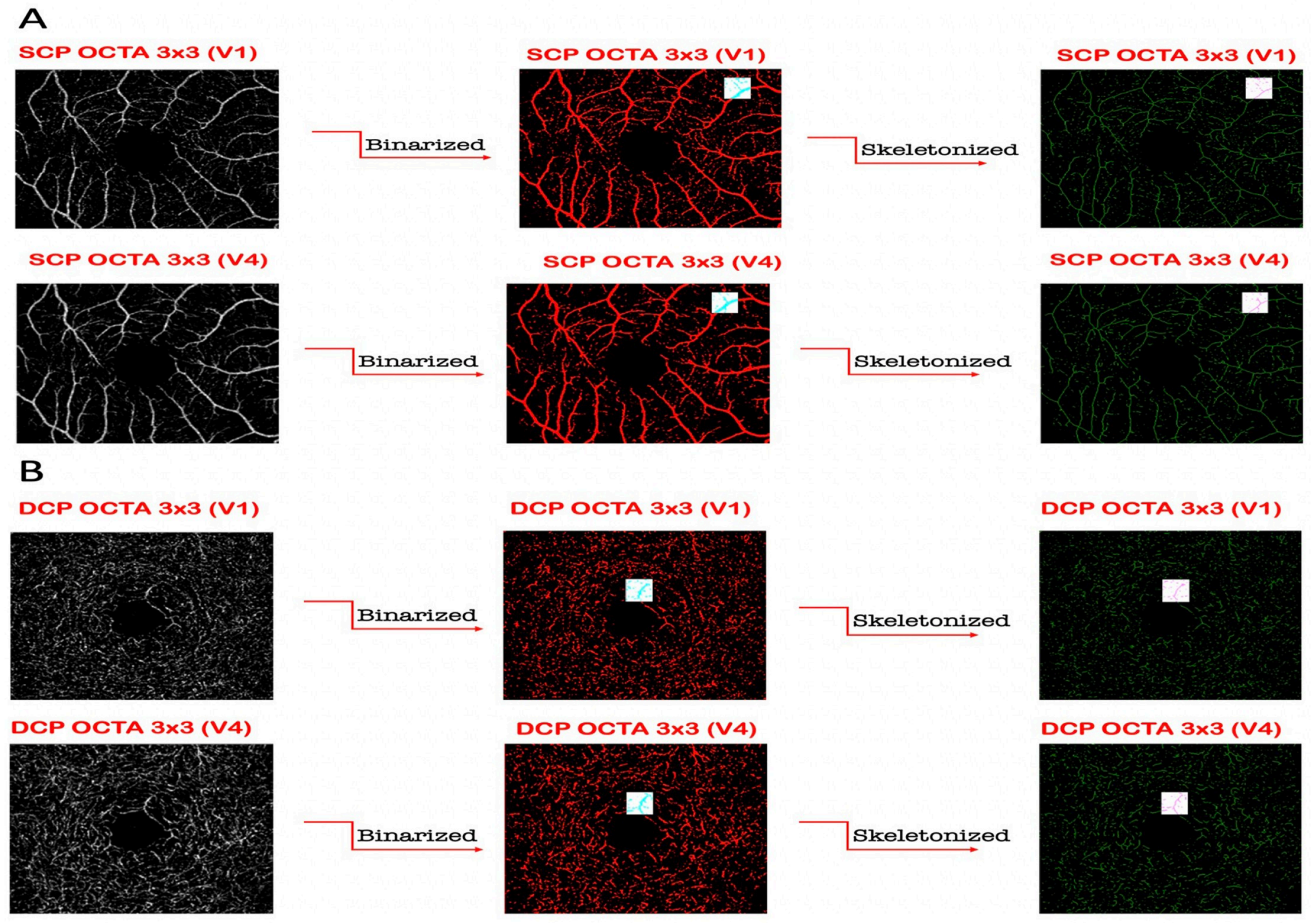
Panel A: Representation of the OCTA assessment of the superficial capillary plexus (SCP). The SCP was investigated in two different OCTA scan modalities: (i) 3x3-mm single volume scan (V_1_) (Top left), and (ii) 3x3 multiple-volume scan (V_4_) (bottom left). The SCP binarized (middle) and skeletonized (right) images, for both protocol scans, were analyzed to investigate both perfusion density (PD), and vessel length density (VLD), respectively. Panel B: Representation of the OCTA assessment of the deep capillary plexus (DCP). The DCP was investigated in two different OCTA scan modalities: (i) 3x3-mm single volume scan (V_1_) (top left), and (ii) 3x3 multiple-volume scan (V_4_) (bottom left). The DCP binarized (middle) and skeletonized (right) images, for both protocol scans, were analyzed to investigate both perfusion density (PD), and vessel length density (VLD), respectively.

**Fig 3:**
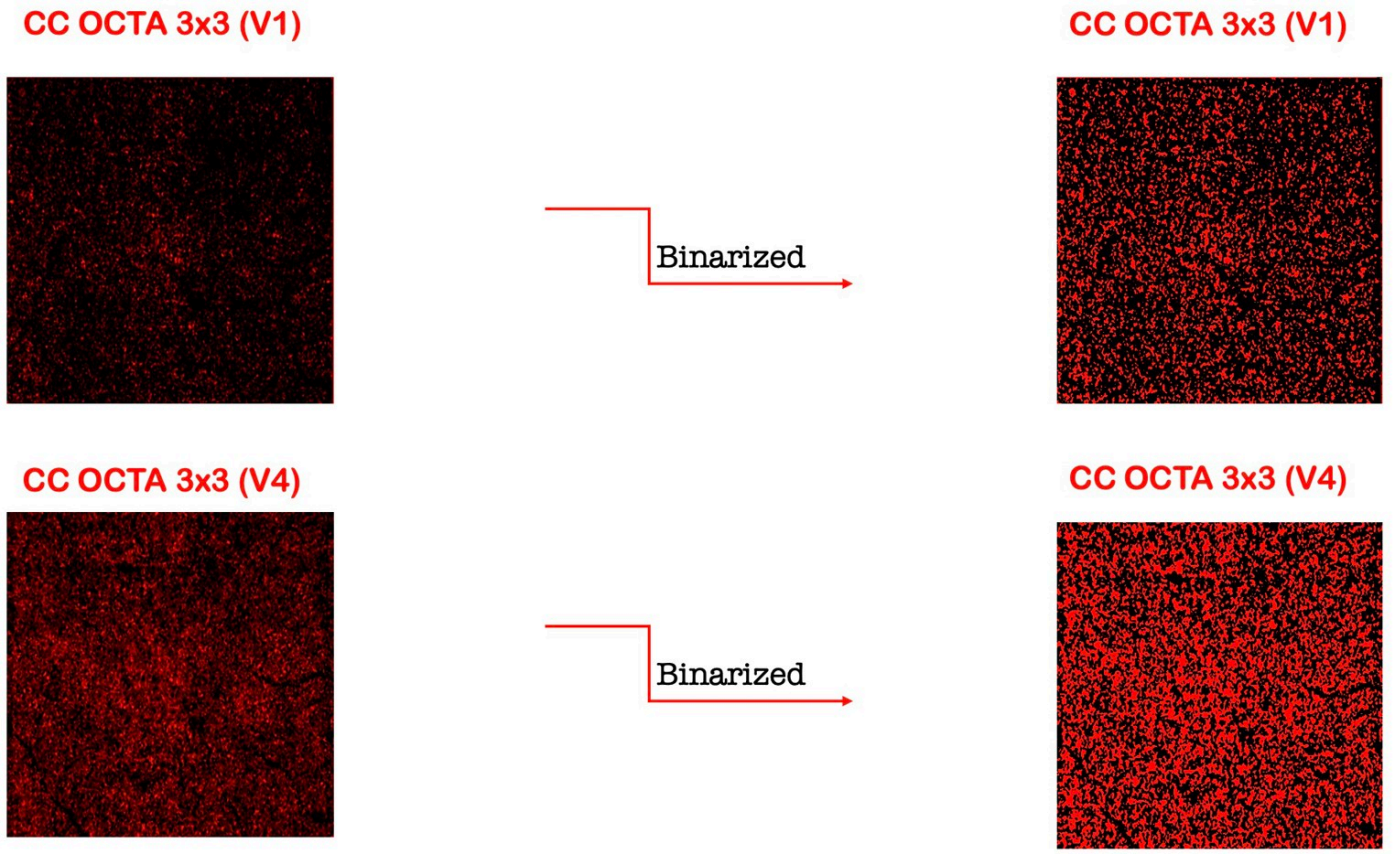
Representation of the OCTA assessment of the choriocapillaris (CC). The CC was investigated in two different scans: (i) 3x3-mm single volume scan (V_1_) (top left), and (ii) 3x3 multiple-volume scan (V_4_) (bottom left). The CC binarized (right) image was analyzed to investigate CC flow deficits.

To quantify these variables, a slight modification of a previously reported algorithm was employed [5, 8–11]. In brief, for each eye, we first exported en face OCTA images (resolution of 304x304 pixels) segmented at the SCP and DCP levels, then they were imported into ImageJ software version 1.50 (National Institutes of Health, Bethesda, MD; available at http://rsb.info.nih.gov/ij/index.html) and consequently were processed with a ‘‘top-hat” filter. Each image was duplicated and two different binarization methods were then performed on the 2 resultant images: (i) 1 image was first processed by a ''hessian'' filter, followed by global thresholding using the ''Huang’s fuzzy'' method; (ii) the other (duplicate) image was binarized using the '' median local'' thresholding. Finally, the two obtained images were combined. The perfusion density (PD) was thus calculated as a unitless proportion of the number of pixels over the threshold divided by the total number of pixels in the analyzed area. Successively, the SCP and DCP images obtained after binarization were skeletonized and these images were employed to measure the vessel length density (VLD) calculated as the total length of the perfused vasculature divided by the total number of pixels in the analyzed area on the skeletonized image (Fig 2) [5, 12]. The vessel diameter index (VDI) which represents the average vessel caliber, was calculated by dividing the total vessel area in binarized image by the total vessel length in the skeletonized image in both SCP and DCP (Fig 2) [9]. Consequently, en face CC images were imported into ImageJ and using automatic local thresholding of the resultant raw data with the Phansalkar method (radius, 15 pixels) it was possible to binarize the CC images (Fig 3). Obtained images were processed with the 'Analyze Particles’ command, to assess the total signal void area and to count and measure the signal voids (CC flow deficits) [13]. The analysis of OCTA imaging measurements was reviewed by two retinal experts (LDA and PV).

### Statistical analysis

All qualitative characteristics of the subjects were summarized as frequency and percentage; quantitative characteristics were summarized as the mean and standard deviation. The reproducibility was evaluated by calculating the concordance correlation coefficient (CCC). The CCC evaluates the degree to which pairs of observations fall on the 45° line through the origin [14]. It contains a measurement of precision ρ (the Pearson correlation coefficient, which measures how far each observation deviates from the best-fit line) and accuracy C_b_ (a bias correction factor that measures how far the best-fit line deviates from the 45° line through the origin): ρc = ρC_b_; in addition, CCC suggests a poor strength of agreement for value below 0.90, moderate from 0.90 to 0.95, substantial from 0.95 to 0.99 and perfect > 0.99 [15]. The Bland-Altman’s method was used to compare the two different techniques (V1 and V4). Bland-Altman’s method consists of plotting the average of the two methods on the x-axis towards the differences between the two methods on the y-axis to evaluate the bias. The bias is, therefore, a systematic error, i.e. the tendency by one of the observers to overestimate or underestimate the variable being measured. It simply quantifies the bias and a range of agreement, within which 95% of the differences between one measurement and the other are included. It is possible to say that the bias is significant when the value zero (line of equality) will not be within its 95% confidence interval. The normal distribution of the data was assessed using the Shapiro-Wilk test. Statistical analysis was performed using MedCalc Statistical Software version 19.5.1 (MedCalc Software bvba, Ostend, Belgium; http://www.medcalc.org; 2019).

## Results

**Fig 4:**
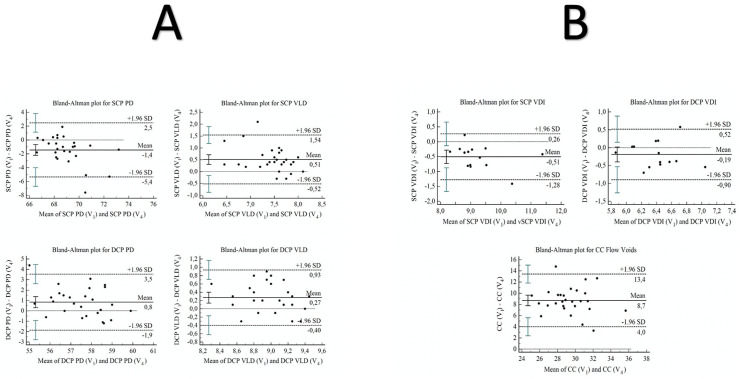
Bland-Altman plots to assess concordance between methods reporting mean between methods for SCP PD, SCP VLD, DCP PD, and DCP VLD variables (A) and SCP VDI, DCP VDI, and CC flow deficits variables (B) reporting on x-axis and differences between methods on the y-axis.

A total of 28 eyes of 28 healthy subjects (12 males and 16 females) aged 26.0 ± 4.0 years were included in this prospective observational cross-sectional study. The demographic characteristics of the study population are reported in Table 1. We found a significant statistical difference in terms of OCTA quantitative metrics by comparing the two different scanning protocols: single volume (V_1_) versus multi-volume merge averages four scan volumes (V_4_). We found a significant difference in PD, VLD, and VDI measurements at the level of both SCP and DCP (Table 2). In detail, the percentage of PD was significantly greater in V_4_ than V_1_, 69.7 ± 2.2 vs 68.2 ± 1.4, respectively (p = 0.005) at the level of SCP. The percentage of VLD was significantly greater in V_1_ compared to V_4_, 7.7 ± 0.4 vs 7.2 ± 0.6 respectively (p<0.001) at the level of SCP. The percentage of PD was significantly greater in V_4_ than V_1_, 57.9 ± 1.2 vs 57.1 ± 1.6 respectively (p = 0.030) at the level of DCP. The percentage of VLD was significantly greater in V_1_ than V_4_, 9.1 ± 0.3 vs 8.8 ± 0.4 respectively (p = 0.004) at the level of DCP. Conversely, VDI measurement resulted significantly greater in V_4_ than in V_1_, 9.5 ± 0.8 vs 9.0 ± 0.7 (p<0.001) for SCP, and 6.5 ± 0.4 vs 6.3 ± 0.3 (p = 0.068) for DCP. The total CC signal void area was significantly greater in V_1_ than to V_4_, 33.7 ± 2.4 vs 25.0 ± 2.7, respectively (p<0.001). The inter-method repeatability of the measurements evaluated by the calculation of CCC showed a poor strength of agreement for all variables considered (Table 3). A Bland-Altman plot was used to assess concordance between methods by reporting mean and differences between methods (Fig 4). We found disagreement between all the measurements except for VDI of DCP (p = 0.068).

**Table 1:** Patient characteristis expressed as mean ± SD or n (column %).

Variable		Mean ± SD or n (Column %)
**SEX**	**Female**	16 (57.1%)
	**Male**	12 (42.9%)
**AGE (years)**		26.0 ± 4.0
**Intraocular pressure (mm Hg)**		16.4 ± 1.5

**Table 2:** Retinal vascular metrics expressed as mean ± SD evaluated for V_1_ (%), V_4_ (%) and differences.

Variable	V_1_ (%)	V_4_ (%)	Δ (V_1_- V_4_)	p-value
**SCP PD**	68.2 ± 1.4	69.7 ± 2.2	-1.4±2.0	0.005
**SCP VLD**	7.7 ± 0.4	7.2 ± 0.6	0.5±0.5	<0.001
**SCP VDI**	9.0 ± 0.7	9.5 ± 0.8	-0.5±0.4	<0.001
**DCP PD**	57.1 ± 1.6	57.9 ± 1.2	0.8±1.4	0.030
**DCP VLD**	9.1 ± 0.3	8.8 ± 0.4	0.3±0.3	0.004
**DCP VDI**	6.3 ± 0.3	6.5± 0.4	-0.2±0.4	0.068
**CC Flow Deficit**	33.7 ± 2.4	25.0 ± 2.7	8.7±2.4	<0.001

**Table 3:** Inter-methods repeatability of measurements: CCC = concordance correlation coefficient.

OCTA (V_1_) vs OCTA (V_4_)	CCC
**SCP PD**	0.308 [0.056 to 0.523]
**SCP VLD**	0.315 [0.084 to 0.513]
**DCP PD**	0.429 [0.148 to 0.645]
**DCP VLD**	0.357 [0.087 to 0.578]
**SCP VDI**	0.728 [0.453 to 0.876]
**DCP VDI**	0.395 [-0.072 to 0.720]
**CC Flow Deficits**	0.080 [0.020 to 0.140]

## Discussion

Since its introduction, OCTA has been widely used, into clinical practice, for imaging and for quantifying retinal microcirculation in healthy [16] and eye diseased [2], and overcoming the use of standard FA [17]. Also, quantitative analyses of OCTA images have the potential to become common use in clinical research settings [18]. Coscas et al firstly reported a normative database to assess vascular density and in superficial and deep capillary plexuses by using a software build-constructed into commercially SD-OCTA device. They showed high repeatability and reproducibility of the measurements [19]. Corvi et al demonstrated that vessel density significantly differs across seven different instruments tested in a cohort of healthy subjects, showing poor reliability among the devices [20]. They recommend the use of the same device to assess the same patient during a clinical setting. That latter can be easily explained because of different algorithms used are not interchangeable nor results readily comparable. Pedinielli and colleagues firstly informed about the impact of different post-processing OCTA imaging by reporting different retinal vessel density values quantified by using three different analytical methods [21]. These results are recently validated by other studies that confirmed the importance to use the same device, same binarization thresholding, as well as OCTA image averaging, to improve retinal functional metric measurements [10, 22]. But, several studies performed OCTA image averaging firstly by recording and exporting multiple images [5], and then by processing them by using external open-source software. It is well known that the combination of more procedures is time-consuming, and can reduce measurement reliability as well as clinical value.

Lauermann et al, firstly speculated that the use of integrated multiple images averaging from on OCTA manufacturer improves image quality parameters [23]. In this prospective observational cross-sectional study, we assessed the quantitative retinal vascular measurements in young healthy subjects. To the author’s knowledge, a comparison of results obtained from two image acquisition modes (not averaged versus multiple averaged OCTA images) by using a novel Ultra-High-Speed SD-OCTA prototype has not been conducted to the date.

We found a significant difference between the measurements obtained by two different image acquisition modes. Particularly, the measurement of perfusion vessel density increased, the measurement of vessel length density decreased, and the measurement vessel diameter index increased using multiple averaged OCTA volume as compared with unaveraged, on both superficial and deep capillary plexuses. Furthermore, we found a decreased percentage of choriocapillaris total flow deficits area measured by using multiple averaged OCTA volume data as compared with unaveraged. These results are in agreement with those reported by other studies [5, 23]. Uji et al speculated that improvement in image quality, by increasing the signal-to-noise ratio, could impact the quantitative analysis from en face OCTA images [5]. It has been widely established that a higher background noise level could reduce the thresholding level for binarization. Thus, multiple en face OCTA image averaging is more reliable than a single image for the assessment of retinal functional parameters. Moreover, the averaged image was rated to have more intensity signal and better continuity of retinal vessels in both SCP and DCP, as well as the evaluation of CC microcirculation that closely resembled the histology [24]. Previous findings suggested that the benefit of image averaging is largely obtained from three acquisitions [5]. Schmidt et al proposed an averaging of ten-volume OCTA frames. Their study determined limited benefit in acquiring and averaging more than five frames [4]. It is well known that increasing the number of images may improve data quantification, but it requires more acquisition time. In our study, we performed four volumetric OCT data sets each of 5 seconds, for a total acquisition time of about 20 seconds. An acceptable, and fast scanning time if compared to the previous study that reported a mean acquisition time of about 29 seconds [23] or more, and apparently less time-consuming if compared to standard FA [17]. Another consideration to point out is that an automated fully-integrated averaging image system appears to be less suffering by misalignments between the registered frames [5, 24]. These latter phenomena may erroneously enlarge the vessel caliber, which in turn could not corroborate the quantification of vascular parameters. Our study has several limitations. First, we reported on a small sample size with 28 subjects. Second, another limitation is that we did not verify the validity of the OCTA scans through the intrasession repeatability calculation, although it has been widely reported by means of SSADA algorithm [19]. Third, we enrolled only young subjects in a relatively narrow age range. It is well known as vascular biomarkers could change with age. Fourth, this study was limited to healthy eyes, accordingly, the examination time may be shorter than in older patients or patients with poor fixation due to maculopathies. Further studies should be addressed to assess the reliability of quantitative measurements by means of automated averaging multiple OCTA images in subjects affected by retinal vascular disease. Fifth, the acquisition of multiple en face OCTA images is more time-consuming compared to the single image. This latter aspect may have implications for daily and busy clinical practice. In summary, our findings showed a significant difference in quantitative measurements between the single and averaged images. PD and VLD have been reported to be clinically relevant quantitative metrics from OCTA imaging. The increased PD and the decreased VLD of both retinal plexuses have highlighted that noise and the evident vessel discontinuity in a single image have a significant impact on these quantitative parameters.

In addition, it does not seem surprising that increasing of VDI values were more significant in SCP than DCP. Indeed, as previously discussed, the benefits of averaging are due to the background noise reduction, and to improvement of vessel continuity which become smooth and of uniform caliber, showing statistically significant values only on the SCP.

In particular, we hypothesize that the significant difference for SCP is due to greater exposure of this plexus being composed of large vessels that affected more by the average system. On the contrary, the DCP is certainly a denser plexus and composed by homogeneous thin capillary vortex converging radially toward an epicenter. This could explain why there is not significant difference for this latter plexus in terms of VDI.

Finally, the decrease of CC flow deficit confirms our results allowing to obtain a CC morphological model very similar to the histologically observed reticulum and might allow the generation of more precise quantitative metrics. Although these promising results, obtained by using a new OCTA device, are in agreement with previous reports, further validation studies are needed, before they can be widely used.
